# Carcinome cuniculatum de la plante

**DOI:** 10.11604/pamj.2016.24.92.8468

**Published:** 2016-05-27

**Authors:** Wassila Boumlil, Fouzia Hali

**Affiliations:** 1 Service de Dermatologie, CHU Ibn Rochd, Université Hassan II, Casablanca, Maroc

**Keywords:** Carcinome, épidermoïde, histologie, exérèse, Carcinoma, epidermoid, histology, excision

## Image en médecine

Le carcinome cuniculatum est un carcinome épidermoïde bien différencié, de bas grade de malignité, rare, qui siège électivement sur le membre inférieur et essentiellement sur la plante du pied (89%). Il est l'apanage du sujet âgé, de sexe masculin. Sa pathogénie reste méconnue. La présentation clinique est évocatrice, mais la confirmation histologique nécessite souvent des biopsies multiples et profondes voire une étude histologique de la pièce d'exérèse. Le principal diagnostic différentiel est la verrue plantaire. Le traitement de choix reste l'exérèse chirurgicale. Aucune récidive n'a été décrite après exérèse complète. Le pronostic est lié essentiellement à l'extension locorégionale. Nous rapportons le cas d'un patient âgé de 50 ans, sans antécédents pathologiques notables, qui consulte pour une tumeur végétante du talon droit, survenant sur un durillon plantaire manipulé et auto-médique, évoluant depuis l’âge de 15 ans dans un contexte de conservation de l’état général. L'examen clinique trouvait une tumeur talonnière bourgeonnante, exophytique, douloureuse, et saignante, de contour régulier, mesurant 10 cm de grand axe. Il s'y associe des durillons plantaires bilatéraux, sans adénopathies notables. Une biopsie cutanée initiale était en faveur d'une verrue vulgaire. Une exérèse complète de la lésion était réalisée. L’étude anatomopathologie de la pièce d'exérèse était en faveur d'un carcinome épidermoïde bien différencié. Le bilan d'extension locorégionale et à distance était négatif. Un recouvrement par lambeau du talon droit, ainsi qu'un traitement médical adéquat des autres durillons plantaires ont été préconisé pour le malade.

**Figure 1:**
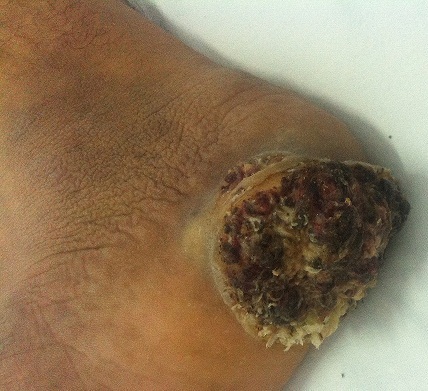
Lésion exophytique du talon droit

